# Lessons from the swamp: developing small molecules that confer salamander muscle cellularization in mammals

**DOI:** 10.1186/s40169-017-0143-8

**Published:** 2017-03-22

**Authors:** JungIn Um, Da-Woon Jung, Darren Reece Williams

**Affiliations:** New Drug Targets Laboratory, School of Life Sciences, Gwangju Institute of Science and Technology, 1 Oryong-Dong, Buk-Gu, Gwangju, 61005 Republic of Korea

**Keywords:** Limb regeneration, Salamander, Small molecules, Cellularization

## Abstract

The ability of salamanders, such as newts, to regenerate damaged tissues has been studied for centuries. A prominent example of this regenerative power is the ability to re-grow entire amputated limbs. One important step in this regeneration process is skeletal muscle cellularization, in which the muscle fibers break down into dedifferentiated, mononuclear cells that proliferate and form new muscle in the replacement limb. In contrast, mammalian skeletal muscle does not undergo cellularization after injury. A significant proportion of research about tissue regeneration in salamanders aims to characterize regulatory genes that may have mammalian homologs. A less mainstream approach is to develop small molecule compounds that induce regeneration-related mechanisms in mammals. In this commentary, we discuss progress in discovering small molecules that induce cellularization in mammalian muscle. New research findings using these compounds has also shed light on cellular processes that regulate cellularization, such as apoptotic signaling. Although formidable technical hurdles remain, this progress increases our understanding of tissue regeneration and provide opportunities for developing small molecules that may enhance tissue repair in humans.

For centuries, scientists have been both fascinated and beguiled by the regenerative capacity of animals such as flatworms, starfish and salamanders [1, 2]. Among these species, salamanders can be thought of as the ‘champions of regeneration’ because they are tetrapod vertebrates that can completely regenerate multiple tissues, such as the lens, ventricle and limb, and partially regenerate their intestine and spinal cord ([3–5], two examples of salamander species are shown in Fig. 1a–b). Characterizing the cellular and molecular mechanisms underlying limb regeneration responses have been the subject of high profile research (for example, [6–9]), with the aim of identifying genes or developing strategies to confer this ability to humans. Much progress has been made in characterizing the signals and cellular responses that initiate and guide these regeneration processes, although our knowledge remains incomplete. However, it has become clear that there are fundamental differences in the initial response of mammals and salamanders to injuries such as limb amputation. One difference is the cellularization of skeletal muscle in salamanders, which contributes dedifferentiated cells to a zone of regenerative cells termed the blastema (described below). In the context of limb regeneration, this had led to the development of small molecules that can change the behavior of mammalian muscle tissue in vitro to resemble the injury response observed in salamanders. In this commentary, we summarize recent progress in the characterization of these small molecules and discuss their potential to be developed as therapeutic agents to enhance tissue regeneration in humans. Moreover, we also describe recent advances in the identification of new drug targets that could be used for the screening and development of compounds that enhance regeneration responses.

**Fig. 1:**
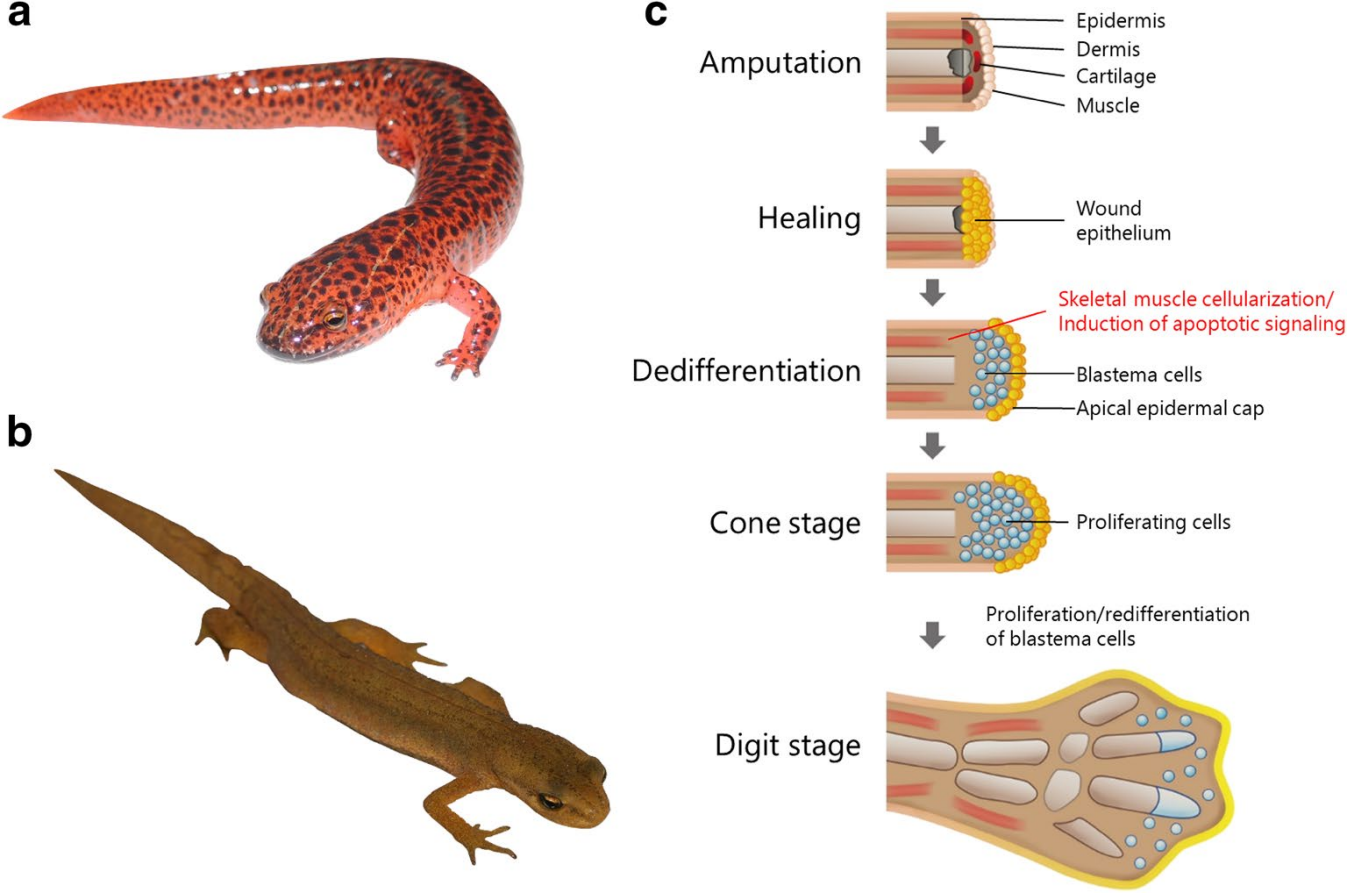
**a**–**b** Examples of two salamander species: the Northern red salamander (*Pseudotriton ruber*) and smooth newt (*Triturus vulgaris*). Most salamander species are between 10 and 20 cm in length [38]. **c** Role of muscle fiber cellularization in limb regeneration. After catastrophic limb injury, such as amputation, a wound epithelium is formed. Skeletal muscle fibers beneath the epithelium lose contact with each other and contract. Apoptotic signaling is initiated and the fiber undergo cellularization, which involves cell cycle re-entry and dedifferentiation. The proliferating mononuclear cells contribute to a zone of proliferating cells termed the blastema, which will form the tissues of the replacement limb. Cellularized skeletal muscle fibers retain memory of their tissue origin and re-differentiate into skeletal muscle in the new limb (Adapted from [39])

There are fundamental differences in the cellular response of salamanders and mammals to limb amputation (discussed in [10]). In mammals, there is simple closure of the wound, followed by healing and scarring. In salamanders, wound closure also occurs with the formation of a blood clot at the site of injury. However, within 6–12 h post-amputation, epidermal cells from the limb stump start to migrate and eventually cover the entire wound surface, forming a structure termed the wound epithelium (Fig. 1c). This epithelium proliferates to form an apical epidermal cap. The epidermis also synthesizes retinoic acid, which is produced as a gradient across the proximal–distal axis of the blastema to provide positional identity [11–13]. The cells in the tissues beneath the wound cap begin to dedifferentiate, including multinucleated skeletal muscle fibers, which break down into single, proliferating cells via the process of cellularization (Fig. 1c). Thus, the limb tissues beneath the amputation site revert to a mass of dedifferentiated cells termed the blastema [14]. A significant proportion of blastema cells originate from multinucleated skeletal muscle fibers in the limb stump [15, 16]. The blastema cells proliferate and re-differentiate over a period of weeks to produce the tissues of the regenerating limb [17, 18]. Blastema cells that originated from cellularized muscle fibers retain memory of their tissue of origin and only form musculature in the regenerating limb [19]. This dedifferentiation does not occur in mammalian skeletal muscle after amputation, suggesting that cellularization is an important step in limb regeneration.

An interesting feature of muscle fiber cellularization is that it can be modelled in vitro using skeletal muscle myotubes [20]. Genetic manipulations or exogenous agents that induce myotube fragmentation can be observed using microscopy and provide a simple assay for candidate compounds that produce cellularization in mammalian muscles [21]. The development of combinatorial chemistry in the 1990s provided small molecule libraries based on known bioactive molecules (in the order of thousands or tens of thousands [22]) that could be screened for inducers of cellularization.

The first reported success using this approach was the discovery of myoseverin (Table 1), which produced cellularization in myotubes derived from the mouse C2C12 myoblast cell line [23]. Myoseverin was found to bind tubulin in the myotubes and destabilize the cytoskeletal network. However, in contrast to other known tubulin binding molecules, myoseverin also increased the expression of genes related to the wound healing response. Subsequent analysis showed that myoseverin down-regulated the myogenic transcription factor, Myf5, in salamander myotubes, which is also observed during the early stages of cellularization [24]. However, further studies of myoseverin using single cell analysis indicated that this compound may only produce myotube fragmentation [25]. The mononuclear cells did not proliferate and remained refractory. Work from our own laboratory showed that further manipulation was required to produce cellularization, such as down-regulation of the cell cycle inhibitor, p21^Cip1^ [26]. Therefore, myoseverin should be categorized alongside other tubulin-binding molecules that cause myotube fragmentation, such as taxol, colchicine and nocodazole. Nevertheless, myoseverin has been useful in the development of small molecule cocktails to induce cellularization (described below).

**Table 1:** Small molecules used to induce cellularization, a key step of salamander limb regeneration, in mammalian myotubes

Structure	Name	Myotube type	Reference	Mechanism	Notes
	BpV [dipotassium bisperoxo (5-hydroxypyridine-2-carboxyl) oxovanadate (V)]	Primary mouse myotubes	[29]	Tyrosine phosphatase inhibitor	10 µM; used in combination with staurosporine and Q-VD
	BIO (6-bromoindirubin-3-oxime)	Mouse C2C12 myotubes and primary mouse myotubes	[33]	Glycogen synthase kinase-3β inhibitor	2.5 µM; used in combination with myoseverin and reversine
	DIDS (5-isothiocyanato-2-[2-(4-isothiocyanato-2-sulfophenyl)ethenyl]benzene-1-sulfonic acid)	Mouse C2C12 myotubes	[35]	Voltage-dependent anionic channel blocker	100 µM; used in combination with staurosporine and Q-VD/Z-VAD
	Lysopho-phatidic acid	Mouse C2C12 myotubes	[33]	G-protein-coupled receptor activator	30 µM; used in combination with myoseverin and reversine
	Myoseverin [9-isoproyl-*N*2,*N*6-bis-(4-methoxybenxyl)-9*H*-purine-2,6-diamine]	Mouse C2C12 myotubes and primary mouse myotubes	[33, 35]	Microtubule disruption	20 µM; used in combination with BIO/lysopho-phatidic acid/SB203580/SQ22536 and reversine
	Q-VD (*N*-(2-quinolyl)-l-valyl-l-aspartyl-(2,6-difluorophenoxy) methylketone)	Mouse C2C12 myotubes and primary mouse myotubes	[29, 35]	Pancaspase inhibitor	10 µM; used in combination with staurosporine and BpV
	Reversine [2-(4-morpholinoanilino)-6-cyclohexylaminopurine]	Mouse C2C12 myotubes and primary mouse myotubes	[33]	Aurora kinase inhibitor	250 nM; used in combination with myoseverin and BIO/lysopho-phatidic acid/SB203580/SQ22536
	SB203580 [4-(4-fluorophenyl)-2-(4-methylsulfinylphenyl)-5-(4-pyridyl)-1*H*-imidazole]	Mouse C2C12 myotubes	[33]	p38 MAP kinase inhibitor	10 µM; used in combination with myoseverin and reversine
	Staurosporine	Mouse A1/C2C12 myotubes and primary mouse myotubes	[35]	ATP-competitive kinase inhibitor	1 µM; used in combination with DIDS/and Q-VD/Z-VAD
	SQ22536 [9-(tetrahydro-2-furanyl]-9*H*-purin-6-amine)	Mouse C2C12 myotubes	[33]	Adenylyl cyclase inhibitor	300 µM; used in combination with myoseverin and reversine
	Z-VAD [*N*-benzyloxycarbonyl-Val-Ala-Asp(O-Me)]	Mouse C2C12 myotubes	[35]	Pancaspase inhibitor	10 µM; used in combination with staurosporine and DIS

Numerous genetic manipulations have been shown to produce cellularization in mammalian myotubes, such as down-regulation of retinoblastoma protein (Rb) and Ink4a/alternative reading frame (ARF) [27], ectopic expression of Msx-1 [9], or overexpression of Twist [28]. This suggests that small molecules could be developed that modulate these targets in mammalian myotubes. The response of mammalian muscle to injuries such as amputation is fiber death rather than regeneration. Paliwal and Conboy [29] speculated that BpV, a tyrosine phosphatase inhibitor that delays myoblast differentiation and increases proliferation, could be used to produce myotube cellularization. However, cell cycle reentry in myotube nuclei has been linked with apoptosis [30]. Therefore, Paliwal and Conboy combined BpV treatment with the apoptosis inhibitor, Q-VD (Table 1). Using genetically labelled mouse myotubes that express yellow fluorescent protein, they observed that the combined chemical treatment induced cellularization, with approximately 10–15% of the myotube nuclei reverting to proliferating mononucleated cells. Marker gene analysis indicated that these cells resembled myoblasts, with increased expression of the myogenic factors Pax7 and MyoD. After transplantation into sites of skeletal muscle damage, the cells re-differentiated into muscle fibers, providing further evidence that combined treatment with BpV and Q-VD induced reversible cellularization in mammalian myotubes. This study provided the first evidence that compound treatment could induce a prominent step of salamander limb regeneration (cellularization) in mammalian muscle tissue.

A critical step in the cellularization process is the initiation of proliferation in the nuclei of the differentiated myotube. It can be hypothesized that small molecules which have been previously shown to induce proliferation in differentiated mammalian cells, such as cardiomyocytes, could be combined with myotube fragmentation compounds to achieve cellularization. In our laboratory, we tested the compound, BIO (Table 1), an inhibitor of glycogen syntheses kinase-3β (GSK-3β), which was previously shown to increase proliferation in refractory mammalian cardiac muscle cells [31, 32]. Sequential treatment of mouse primary myotubes with myoseverin and BIO induced cellularization [33]. Step-wise treatment with the small molecule, reversine, an epigenetic regulator that produces dedifferentiation [34], induced pluripotent potential in the mononuclear cells as indicated by re-differentiation into the neuronal lineage. The cell cycle inhibitor, p21^Cip1^ is known as a ‘gatekeeper’ that maintains skeletal muscle differentiation. Using three different small molecules that have been shown to down-regulate p21 expression or stability; lysophosphatidic acid (activator of G-protein-coupled receptors), SQ22536 (adenylyl cyclase inhibitor) and SB203580 (p38 MAP kinase inhibitor) (Table 1) our laboratory demonstrated that these molecules can induce cellularization in mouse C2C12 myotubes when combined with myoseverin treatment [33]. This suggests that different signaling pathways can be manipulated to induce cellularization if they converge on a common target, such as p21^Cip1^.

Recently, new insights about the role of chemically-induced apoptosis in cellularization were reported [35]. Mammalian myotube fragmentation using myoseverin was shown to induce apoptosis in the mononuclear cells. Using the well-known apoptosis-inducing compound, staurosporine (Table 1), it was observed that the induction of apoptosis is a critical event in myotube fragmentation. However, to prevent cell death and achieve cellularization, the apoptotic process should be ‘intercepted,’ which was achieved using the anti-apoptotic small molecules DIDS (inhibitor of voltage-dependent anionic channels) and Z-VAD or Q-VD (pancaspase inhibitors) (Table 1) [35]. Significantly, similar processes were revealed in regenerating salamander limb tissues. Tracing the activity of the apoptosis ‘executioner’ cleaved caspase-3 showed that blastema cells maintained caspase activity without undergoing apoptosis. Therefore, sequential treatment with staurosporine and DIDS/Q-VD produces cellularization in mammalian myotubes, which mimics the initiation and suppression of apoptosis pathways observed in the regenerating salamander limb.

Table 1 provides a list of the small molecules that have been used to induce cellularization in mammalian myotubes. Currently, these molecules are only effective in combination: there is no reported single molecule treatment for producing cellularization. An interesting feature is that, even though numerous genetic manipulations have been shown to achieve cellularization, such as over-expression of Msx-1 or down-regulation of Rb/ARF [9, 27], these molecules target different proteins, such as tubulin or GSK-3β. Therefore, there is ample scope to develop new small molecules that achieve cellularization by targeting known genetic regulators.

In summary, significant progress has been achieved in the development of small molecules that produce a critical step of limb regeneration: cellularization in mammalian muscle tissue. Recent research indicates that apoptotic signaling without full progression to apoptosis is a significant stage of regeneration in salamanders. Therefore, focusing on small molecules that induce apoptosis in muscle, such as doxorubicin [36], could provide new candidate compounds for initiating cellularization. However, it is also apparent that major technical hurdles remain before there is any possibility of completely regenerating mammalian limb tissues. For example, the cellularization process has been recapitulated in mammalian myotubes, but there is no data to suggest that this can be reproduced in fully differentiated mature muscle fibers, which are thicker and striated with contractile proteins. Moreover, small molecule-induced cellularization has only been reported for mouse myotubes. There is no report about the effectiveness of this methodology in human muscle tissue. Finally, there is no established animal model for testing the potential for these small molecules to enhance tissue regeneration in vivo. The p21^Cip1^ knockout mouse shows appendage regeneration with the closure of ear punctures [37]. It could be envisaged that small molecules which induce myotube cellularization by targeting p21^Cip1^ could be tested in an ear punch model using genetically normal mice. Overall, numerous small molecules have been developed that induce a key step of salamander limb regeneration, cellularization, in mammalian muscle tissue. Unfortunately, research progress is stalled at in vitro analyses of myotubes derived from rodent tissues. Further studies of these molecules in vivo using animal models and confirmation of their effects in human tissues are required to assess their potential to regenerate tissues that have been lost to injury or disease.

## References

[1] Tanaka EM, Reddien PW (2011). The cellular basis for animal regeneration. Dev Cell.

[2] Dubois P, Ameye L (2001). Regeneration of spines and pedicellariae in echinoderms: a review. Microsc Res Tech.

[3] O’Steen WK, Walker BE (1962). Radioautographic studies or regeneration in the common newt. III. Regeneration and repair of the intestine. Anat Record.

[4] Egar M, Singer M (1972). The role of ependyma in spinal cord regeneration in the urodele. Triturus Exp Neurol.

[5] Odelberg SJ (2005). Cellular plasticity in vertebrate regeneration. Anat Record Part B New Anat.

[6] Kumar A, Godwin JW, Gates PB, Garza-Garcia AA, Brockes JP (2007). Molecular basis for the nerve dependence of limb regeneration in an adult vertebrate. Science.

[7] Sugiura T, Wang H, Barsacchi R, Simon A, Tanaka EM (2016). MARCKS-like protein is an initiating molecule in axolotl appendage regeneration. Nature.

[8] Nacu E, Gromberg E, Oliveira CR, Drechsel D, Tanaka EM (2016). FGF8 and SHH substitute for anterior–posterior tissue interactions to induce limb regeneration. Nature.

[9] Odelberg SJ, Kollhoff A, Keating MT (2000). Dedifferentiation of mammalian myotubes induced by msx1. Cell.

[10] Brockes JP, Kumar A (2008). Comparative aspects of animal regeneration. Annu Rev Cell Dev Biol.

[11] Maden M (1982). Vitamin A and pattern formation in the regenerating limb. Nature.

[12] Stocum DL, Crawford K (1987). Use of retinoids to analyze the cellular basis of positional memory in regenerating amphibian limbs. Biochem Cell Biol.

[13] Brockes JP (1992). Introduction of a retinoid reporter gene into the urodele limb blastema. Proc Natl Acad Sci USA.

[14] Karczmar AG (1947). The effect of repeated amputation and blastema formation on regeneration. Anat Record.

[15] Thorton CR (1938). The histogenesis of muscle in the regenerating forelimb of larval *Amblystoma* punctatum. J Morph.

[16] Hay ED (1959). Electron microscopic observations of muscle dedifferentiation in regenerating *Amblystoma* limbs. Dev Biol.

[17] Spallanzani A (1769). Reproductions of the legs in the aquatic salamander. An essay on animal reproductions.

[18] Hay ED, Fischman DA (1961). Origin of the blastema in regenerating limbs of the newt *Triturus viridescens*. An autoradiographic study using tritiated thymidine to follow cell proliferation and migration. Dev Biol.

[19] Kragl M, Knapp D, Nacu E, Khattak S, Maden M, Epperlein HH (2009). Cells keep a memory of their tissue origin during axolotl limb regeneration. Nature.

[20] Capers CR (1960). Multinucleation of skeletal muscle in vitro. J Biophys Biochem Cytol.

[21] Bischoff R, Holtzer H (1968). The effect of mitotic inhibitors on myogenesis in vitro. J cell Biol.

[22] Schreiber SL (1998). Chemical genetics resulting from a passion for synthetic organic chemistry. Bioorg Med Chem.

[23] Rosania GR, Chang YT, Perez O, Sutherlin D, Dong H, Lockhart DJ (2000). Myoseverin, a microtubule-binding molecule with novel cellular effects. Nat Biotechnol.

[24] Imokawa Y, Gates PB, Chang YT, Simon HG, Brockes JP (2004). Distinctive expression of Myf5 in relation to differentiation and plasticity of newt muscle cells. Int J Dev Biol.

[25] Duckmanton A, Kumar A, Chang YT, Brockes JP (2005). A single-cell analysis of myogenic dedifferentiation induced by small molecules. Chem Biol.

[26] Jung DW, Williams DR (2011). Novel chemically defined approach to produce multipotent cells from terminally differentiated tissue syncytia. ACS Chem Biol.

[27] Pajcini KV, Corbel SY, Sage J, Pomerantz JH, Blau HM (2010). Transient inactivation of Rb and ARF yields regenerative cells from postmitotic mammalian muscle. Cell Stem Cell.

[28] Hjiantoniou E, Anayasa M, Nicolaou P, Bantounas I, Saito M, Iseki S (2008). Twist induces reversal of myotube formation. Differ Res Biol Divers.

[29] Paliwal P, Conboy IM (2011). Inhibitors of tyrosine phosphatases and apoptosis reprogram lineage-marked differentiated muscle to myogenic progenitor cells. Chem Biol.

[30] Endo T, Nadal-Ginard B (1998). Reversal of myogenic terminal differentiation by SV40 large T antigen results in mitosis and apoptosis. J Cell Sci.

[31] Tseng AS, Engel FB, Keating MT (2006). The GSK-3 inhibitor BIO promotes proliferation in mammalian cardiomyocytes. Chem Biol.

[32] Kim WH, Shen H, Jung DW, Williams DR (2016). Some leopards can change their spots: potential repositioning of stem cell reprogramming compounds as anti-cancer agents. Cell Biol Toxicol.

[33] Kim WH, Jung DW, Kim J, Im SH, Hwang SY, Williams DR (2012). Small molecules that recapitulate the early steps of urodele Amphibian Limb regeneration and confer multipotency. ACS Chem Biol.

[34] Chen S, Zhang Q, Wu X, Schultz PG, Ding S (2004). Dedifferentiation of lineage-committed cells by a small molecule. J Am Chem Soc.

[35] Wang H, Loof S, Borg P, Nader GA, Blau HM, Simon A (2015). Turning terminally differentiated skeletal muscle cells into regenerative progenitors. Nat Commun.

[36] Sardao VA, Oliveira PJ, Holy J, Oliveira CR, Wallace KB (2009). Morphological alterations induced by doxorubicin on H9c2 myoblasts: nuclear, mitochondrial, and cytoskeletal targets. Cell Biol Toxicol.

[37] Bedelbaeva K, Snyder A, Gourevitch D, Clark L, Zhang XM, Leferovich J (2010). Lack of p21 expression links cell cycle control and appendage regeneration in mice. Proc Natl Acad Sci USA.

[38] Stebbins RC, Cohen NW (1995). A natural history of Amphibians.

[39] Stewart S, Rojas-Munoz A, Izpisua Belmonte JC (2007). Bioelectricity and epimorphic regeneration. BioEssays News Rev Mol Cell Dev Biol.

